# Endovascular Management of the Ascending Aorta: State of the Art

**DOI:** 10.14797/mdcvj.1173

**Published:** 2023-03-07

**Authors:** Aidan D. Atkins, Michael J. Reardon, Marvin D. Atkins

**Affiliations:** 1 Texas A&M University Department of Biomedical Engineering, College Station, Texas, US; 2 Houston Methodist DeBakey Cardiovascular Surgery Associates, Houston, Texas, US

**Keywords:** ascending aortic stent graft, endovascular ascending repair, ascending aortic dissection, ascending aortic aneurysm

## Abstract

Endovascular stent graft repair (EVAR) has revolutionized the management of aneurysms and dissections of the thoracic and abdominal aorta and is considered the first-line treatment in such pathologies. Initially designed for patients unfit for open repair, EVAR and thoracic endovascular aortic stent graft repair are associated with improved morbidity and mortality and a faster recovery process. The endovascular revolution of the aorta continues moving proximally, with fenestrated and branch stent grafts currently in clinical trials for the management of thoracoabdominal aortic aneurysms, and several branched thoracic devices are either approved or in trial for management of aortic arch pathologies. The final frontier in the endovascular management of the aorta is the aortic root and ascending aorta. The first early feasibility trial for management of type A aortic dissection has recently concluded with a multicenter phase 2 study slated for the spring of 2023. The following article updates the reader on the unique challenges of endovascular management of the ascending aorta and a look at the future technologies that will define this space.

## Introduction

Thoracic endovascular aortic stent graft repair (TEVAR) has revolutionized the management of descending aortic pathologies, showing significant improvements in mortality, morbidity, and recovery time. Even so, open surgical repair via median sternotomy remains the treatment of choice for pathologies involving the ascending aorta, most commonly ascending aortic dissection and degenerative aneurysms. Less frequent pathologies, such as penetrating ulcers, intramural hematoma, and pseudoaneurysms at surgical sites, are sometimes amenable to ascending thoracic stent graft repair. In select patients with anatomy amenable to thoracic stent grafting alone, TEVAR has been used with reasonable success.

In patients whose anatomy is not conducive to open surgical repair, the only treatment options include hybrid procedures (extrathoracic debranching of the brachiocephalic vessels combined with TEVAR) or medical management alone. Extrathoracic debranching combined with TEVAR has had mixed success depending on the pathology type and the patient’s anatomy, but medical management in cases of ascending dissection and aneurysm has typically had poor results. In the International Registry of Aortic Dissection, the “turn down rate” is approximately 10% for open surgical repair in inoperable patients presenting with ascending dissection.^1^ Less invasive treatment options and specific devices for ascending aortic procedures are desperately needed for such patients.

Ascending aortic-specific devices have been slow to develop, with only one phase 1 clinical trial conducted to date. This study examined the use of a specifically designed stent graft in high-risk patients presenting with acute type A aortic dissection and showed promising results.^2^ A phase 2 clinical trial is planned for 2023. One custom ascending specific device has been used on a limited basis in Europe but is no longer being manufactured. A second custom ascending device is in early feasibility testing in Europe.

The ascending aorta and aortic root are the “final frontier” in the endovascular revolution of aortic pathologies. This review focuses on the endovascular management of ascending aortic pathologies and describes how their unique anatomical and physiological characteristics present a challenge for successful management.

## Background

Endovascular stent graft therapy has revolutionized the management of aneurysms, dissections, and blunt traumatic aortic injuries involving the descending thoracic aorta since Dake’s original description of its use in 1994.^3^ In May 2005, the first commercially available thoracic stent graft (WL Gore TAG device) was introduced to the market, and since that time we have seen multiple iterations of thoracic stent graft devices from various manufacturers. Newer generations of devices have had lower profiles that allow smaller arterial access, improved delivery systems, and the ability to shape the grafts to conform to the angulation of the distal aortic arch. A few devices also have been removed from the market secondary to stent fractures and rare cases of graft collapse. The first single-branched thoracic stent graft device was approved by the US Food and Drug Administration (FDA) as recently as May 2022.^4^ Several multibranched thoracic stent graft devices are currently undergoing clinical trials in the US for aortic arch pathology. At present, device design and clinical trial efforts of various manufacturers have focused on the aortic arch rather than the ascending aorta and root.

**Figure 1:**
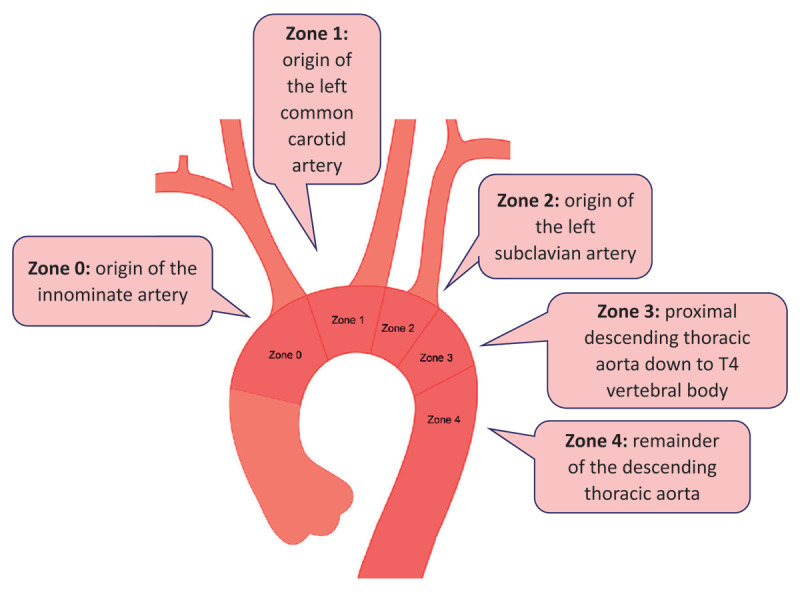
Ishimaru classification scheme with five aortic landing zones.^5^

The Ishimaru classification scheme defines the five TEVAR landing zones for comparing endovascular procedures. Each zone is bordered by a tangential line aligned with the distal edge of each great vessel, including: zone 0, the origin of the innominate artery; zone 1, the origin of the left common carotid artery; zone 2, the origin of the left subclavian artery; zone 3, the proximal descending thoracic aorta down to T4 vertebral body; and zone 4, the remainder of the descending thoracic aorta. This review focuses on zone 0 and the ascending aorta (Figure 1).^5^

Given the improved clinical outcomes in the endovascular management of descending thoracic aorta pathologies, this approach has been increasingly applied in specific circumstances to the ascending aorta. The limiting anatomic factor in using commercially available thoracic stent grafts in the ascending aorta has typically been their length. The shortest thoracic stent graft devices are typically 10 cm, and the usual anatomic distance from the sinotubular junction to the innominate artery is typically less than 10 cm, which precludes their use. Surgeons also have used abdominal aortic stent graft cuffs off-label in the ascending aorta, but typically the delivery shaft is not long enough to reach from a transfemoral approach; these devices have been placed from a transapical approach or via the axillary, subclavian, or carotid arteries. Multiple overlapping devices, when needed, predispose to endoleaks (ie, type 3 endoleak) between the junctions. Such devices work reasonably well in isolated aortic pathologies such as penetrating ulcers and pseudoaneurysms, although their use in degenerative aneurysms or ascending aortic dissections has been very limited, with mixed results.^6^–^9^

Ascending-specific aortic devices are currently being developed by manufacturers. As with most endovascular devices, their initial clinical use occurs outside the US for several years prior to the start of FDA clinical trials because initial feasibility studies can be performed outside of the US for a fraction of the cost prior to committing to a costly US-based trial.

## Ascending Aorta: Anatomic and Physiological Characteristics

A basic understanding of the anatomical characteristics of the ascending aorta and the various ascending aortic pathologies is helpful for a deeper understanding of the role of endovascular management and the unique anatomic and physiologic challenges when developing ascending aortic-specific devices.

The wall of the aorta is composed of three distinct layers: intima, media, and adventitia. The intima is the thinnest layer, consisting of a thin ground substance lined by endothelium, and is easily traumatized. The media is the thickest layer, consisting of elastic fibers arranged in a spiral fashion to increase tensile strength. The adventitia is a thin fibrous layer and contains the vasa vasorum, which carries nutrients to the media. The aorta is highly compliant and expands and contracts during the cardiac cycle due to the elastic fibers in the media. With age, this compliance decreases due to fragmentation of the elastic fibers and an increase in the fibrous tissue of the media. In addition, both hypertension and atherosclerosis cause premature aging of the aorta.

The different segments of the aorta are affected by forces during the cardiac cycle. The aortic root is pulled with every heartbeat and contraction of the ventricles, leading to longitudinal and radial expansion. The ascending aorta is mobile and has significant changes in size during systole. The ascending aorta stores up to 50% of the left ventricular stroke volume during systole, a phenomenon termed the “Windkessel effect.” During diastole, the aorta reduces in diameter due to its elastic properties and moves the blood volume antegrade down the aorta to the branch vessels; it also moves retrograde back towards the aortic root, providing coronary blood flow during diastole. The Windkessel effect converts the pulsatile systolic cardiac flow into a near continuous flow to the remaining segments of the aorta during the entire cardiac cycle. This storage of blood within the ascending aorta significantly reduces cardiac afterload. Stiffness within the aorta wall secondary to aging, atherosclerosis, and hypertension offsets the Windkessel effect and leads to increased cardiac afterload, reduced coronary perfusion pressure during diastole, and increased pulsatile energy transmitted to distal segments of the aorta and its branch arteries.^10,11^

The aortic arch is relatively fixed compared with the ascending aorta due to the brachiocephalic vessels and their attachments to the surrounding mediastinal structures entering the neck. The distal aortic arch is fixed on its undersurface to the pulmonary artery at the ligamentum arteriosum. This is the most frequent site of blunt aortic injury during sudden deceleration. The descending thoracic aorta is also relatively fixed due to the mediastinal pleural and the intercostal branches at every rib space. The elasticity of the aorta decreases distally due to the amount and configuration of the elastic fibers within the media.

Aneurysm and dissection of the ascending aorta are the most frequent pathological processes encountered in the ascending aorta. Degenerative diseases of the media with aneurysm formation are the most common disorders of the aortic root and ascending aorta. Severe degeneration of the media can be seen early in life, in genetic diseases such as Marfan’s syndrome and Loeys-Dietz syndrome. In Marfan’s syndrome, a defect in the *FBN1* gene makes fibrillin, a glycoprotein essential for the formation of elastic fibers of the aorta and in other connective tissues. Loeys-Dietz syndrome is characterized by five separate types distinguished by genetic mutations in transforming growth factor beta or SMAD proteins affecting the signaling pathway leading to elastin production. Bicuspid and unicuspid aortic valves (5% and < 1% of the population, respectively) can also be associated with an aortopathy and dilatation of either the aortic root and/or ascending aorta. Atherosclerosis, aging, and a familial history also may be frequent causes of aneurysms and dissections of the ascending aorta and aortic root.

Aortic dissection of the ascending aorta (frequently seen in the setting of hypertension and/or a previously unknown aneurysm) is a frequent clinical entity. Aortic dissection has classically been divided into proximal and distal dissections (Stanford A & B and DeBakey Classifications I, II, IIIA, and IIIB), with two-thirds of dissections involving the ascending aorta. It is one of the most frequently encountered emergencies in a cardiothoracic surgeon’s practice.

Other unusual pathologies involving the ascending aorta include intramural hematoma, which involves bleeding within the wall of the aorta, possibly from ruptured vasa vasorum vessels in the medial layer and penetrating ulcers. Intramural hematoma typically appears as hyperdense blood within the wall but with no imaging evidence of flow in a false lumen or a point of entry within the intima. Penetrating ulcers typically involve an atherosclerotic plaque that erodes the layers of the aortic wall and can lead to disruption of the outer adventitia and rupture.

## Anatomical Challenges to Endovascular Management of the Ascending Aorta

**Figure 2:**
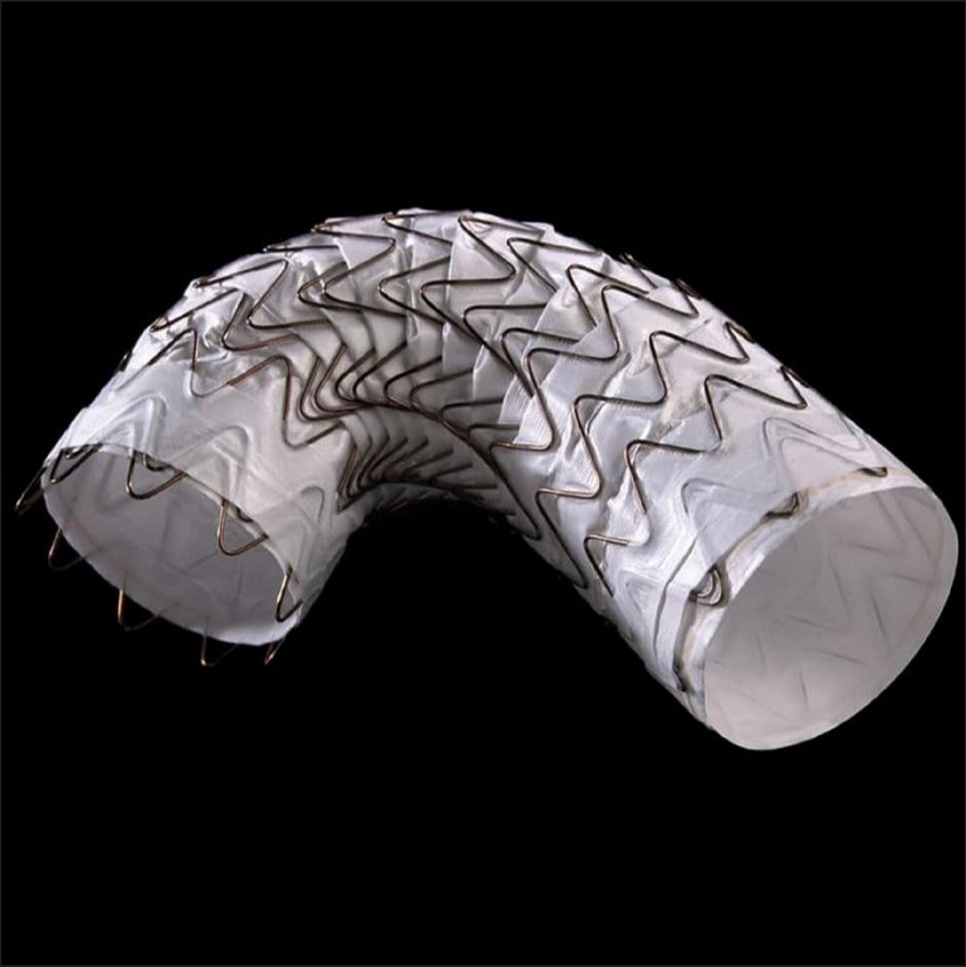
Gore Ascending Stent Graft. Reprinted with permission; GORE® ASCENDING STENT GRAFT © 2023. See Instructions for Use for complete device information, including approved indications and safety information.

The ascending aorta has several anatomical features that add to the challenge of stent grafting. This curved structure has an outer curvature much longer than the inner curvature, making precise stent graft deployment and anatomic fixation difficult. The Gore Ascending Stent Graft has been designed to overcome this with its ability to flex during deployment (Figure 2). Another challenge is aging. The ascending aorta can elongate and angulate as patients’ age, which makes endovascular repair difficult. The length of the ascending aorta varies significantly, and a wide range of device lengths and diameters would need to be available to treat patients, especially on an emergency basis.

The ascending aorta also changes in diameter throughout the cardiac cycle, thus the Windkessel effect is important in left ventricle (LV) unloading and coronary blood flow during diastole. It is currently unclear to what effect (if any) that rigid stent grafting of the ascending aorta has on the Windkessel effect. The materials used in ascending aortic stent graft are either polytetrafluoroethylene, woven, or tightly knitted Dacron fixed to nitinol or stainless-steel stents. These materials do not have the same expansile characteristics as the native ascending aorta, and their effect on aortic remodeling, LV unloading, and coronary blood flow are yet to be determined.

Dacron grafts, used for decades in the management of aortic pathologies, have been found to have four times the reduced compliance of native aortic tissue. This concept of “compliance mismatch” between the graft and native aorta is now thought to have adverse local and downstream effects. There appears to be adverse local effects at the graft/aortic anastomosis leading to anastomotic pseudoaneurysms. This is markedly pronounced when the graft is sutured to abnormal aortic tissue. One clinical example of this would be the creation of a hemiarch anastomosis in the setting of a dilated and/or dissected aortic arch. It is well known that the residual aortic arch is at significant risk for further dilatation. This compliance mismatch at the graft aortic junction may contribute from a flow dynamic standpoint to altered shear stress in the residual aorta, leading to further degeneration. Retrograde adverse effects also appear on the LV due to increased ventricular afterload leading to hypertrophy.

Studies have evaluated patients who have undergone ascending and even thoracoabdominal aortic aneurysm repairs and later developed LV hypertrophy.^12^ Much research is ongoing regarding the elastic-mechanical properties of currently used Dacron and polytetrafluoroethylene grafts. Thoracic stent grafts, typically made from similar materials but with an added external nitinol or stainless-steel skeleton, will likely have similar or possibly even worse compliance mismatch. Further research into the late-term hemodynamic effects of such grafts is needed. The ideal replacement material for the aorta—biomimetic grafts capable of reproducing the same hemodynamic and mechanical characteristics of the native aorta when implanted—do not yet exist.^12^

The pathologies of aortic dissection and aneurysm typically extend down to the sinotubular junction or even further down into the aortic root. Most ascending aortic dissections have the entry tear within 2 cm of the sinotubular junction. A principle of endovascular management of aortic dissections is coverage of the proximal entry tear to stop false lumen flow and induce thrombosis of the false lumen. Many patients presenting with ascending aortic dissection do not have enough “normal” aorta proximal to the entry tear to get a proximal seal. This distance has been debated but is at least 1 to 2 cm from the highest coronary artery to the start of the entry tear. This is one of the most frequent anatomic criteria that excludes patients from studies of ascending aortic dissection devices.

Thoracic stent grafting for degenerative aneurysmal disease typically requires a certain distance of non-aneurysmal, ideally parallel aortic wall, both proximally and distally. In many instances, there is not enough normal proximal aorta for a seal zone, precluding endovascular treatment in degenerative aneurysms.

In patients with a known or suspected connective tissue disorder, stent graft repair has typically been avoided except in dire circumstances in those deemed unfit for open repair. The outward radial force of the stent grafts may lead to late failure of device sealing. In selected patients with a connective tissue disorder, ascending and aortic arch stent grafting has been performed if the devices can seal in previously placed surgical grafts.^13^

## Current Devices for use in the Ascending Aorta

### Single Branch Devices

There are currently multiple single branch devices for aortic arch pathologies undergoing clinical trials in the US. These devices were initially designed for placement in the left subclavian artery in the management of descending aortic pathologies. These devices have been extended proximally to be placed in either the left common carotid artery (combined with a left carotid subclavian bypass) or into the innominate artery (combined with a right-to-left carotid-carotid bypass +/– left carotid subclavian bypass). These devices are covered further in the article, “Branched and Fenestrated Aortic Endovascular Grafts,” in this issue of the *Methodist DeBakey Cardiovascular Journal*.

### Multi-Branch Devices

There are three multi-branch arch devices that have been used in ascending aortic pathologies. The Terumo Aortic Relay-Branch is a two-sided branch device with the proximal graft landing in zone 0. The Cook arch branch graft is a custom device with 2 or 3 inner side-branch portals for aortic arch pathologies, which also extend proximally into the ascending aorta. These devices also are covered further in the article, “Branched and Fenestrated Aortic Endovascular Grafts” in this issue.

## Ascending Specific Devices

### Cook Ascending Graft

**Figure 3:**
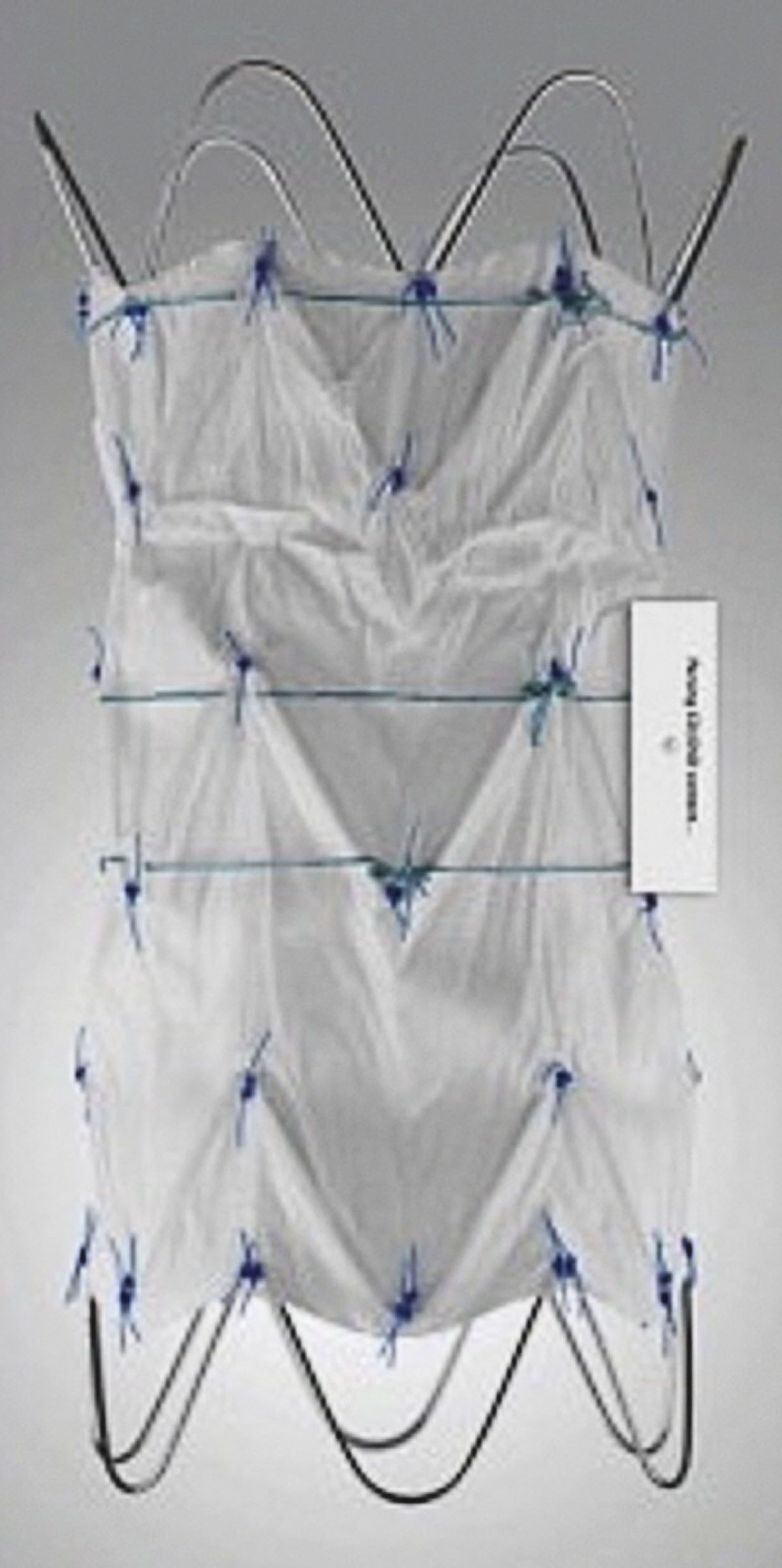
Cook ascending graft, courtesy of Cook Medical.

The Cook ascending graft (Figure 3) was an ascending aortic specific device that had been used internationally for various pathologies of the ascending aorta. This device is no longer manufactured globally. The device is a tubular endograft constructed of woven polyester fabric sewn to self-expanding nitinol stents with braided polyester and monofilament suture. The graft incorporates uncovered nitinol stents both proximally and distally. The device was produced with diameters of 28 mm to 46 mm and a covered length of 6.5 cm. Tsilimparis and colleagues presented their initial experience with this device in 10 patients in a multicenter international registry in 2016.^14^ The clinical success rate was 100% and 30-day survival was 90%. The device was used in five patients with dissection, four with an aneurysm, and one for fixation of a dislocated bioprosthetic aortic valve. Complications included unsuccessful treatment of an intraprocedural TAVR-valve-associated aortic dissection leading to early mortality. Early neurological events included one patient with stroke and paraplegia and another with a transient ischemic attack. At a mean follow-up of 10 months, three late deaths occurred and one was thought to be due to graft infection. Given this cohort of patients deemed unsuitable for open repair, the early experience appeared to be safe and feasible.

Tsilimparis published an updated single center experience with 27 procedures in 24 patients using the Cook ascending graft device.^15^ Indications included acute or chronic ascending dissection (n = 16), pseudoaneurysm (n = 6), fixation of a dislocated TVAR valve (n = 2), and miscellaneous (n = 3). Of the procedures, 17 were performed urgently. Technical success was 100%, the 30-day mortality rate was 21% (only one was thought related to the device), and major stroke in two patients (7.4%). The Cook ascending graft is no longer manufactured globally despite its initial promising experience.

### Relay Custom Medical Device

**Figure 4:**
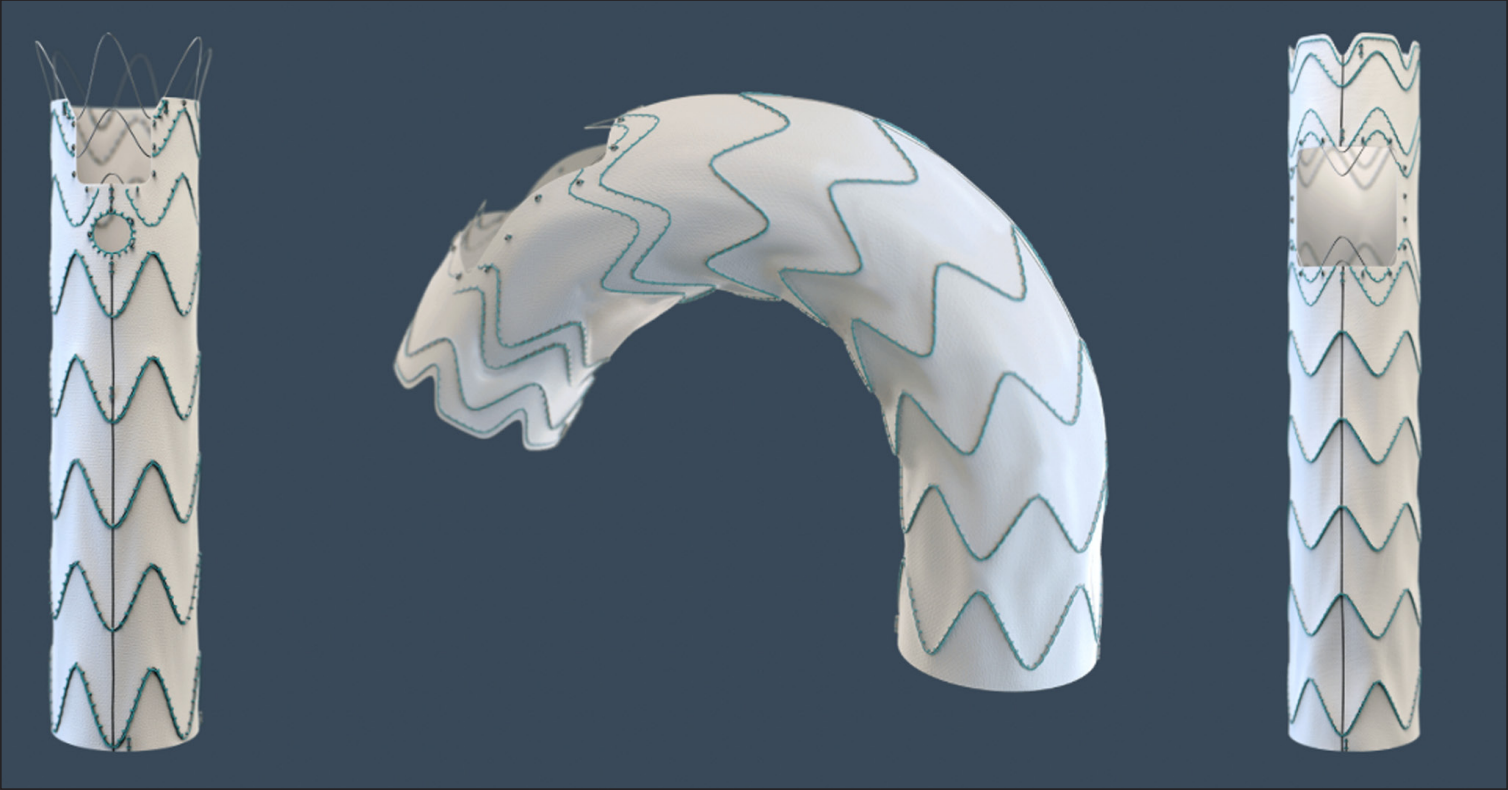
Terumo Aortic Relay Custom Medical Device. Used with permission from Terumo Medical Corporation.

The Relay Custom Medical Device (Terumo Aortic) is a custom-built thoracic stent graft that can be built with the patient’s anatomic specifications within 3 weeks from device design approval. The device can be a straight tube to treat the ascending aorta with a single piece. It also can be built with custom large fenestrations for all of the brachiocephalic vessels, with sealing in the ascending aorta or with scallops and smaller fenestrations to treat aortic arch pathologies (Figure 4). This device is currently not available in the US. Piffaretti et al. published a multicenter experience with the Relay Custom Medical Device in nine patients that included five pseudoaneurysms, three uncomplicated Type A dissections, and one contained rupture.^16^ Clinical success was achieved in all patients, with no operative mortality, no major stroke, and no conversions to open surgery.

### Gore Ascending Stent Graft

#### ARISE Trial

**Figure 5:**
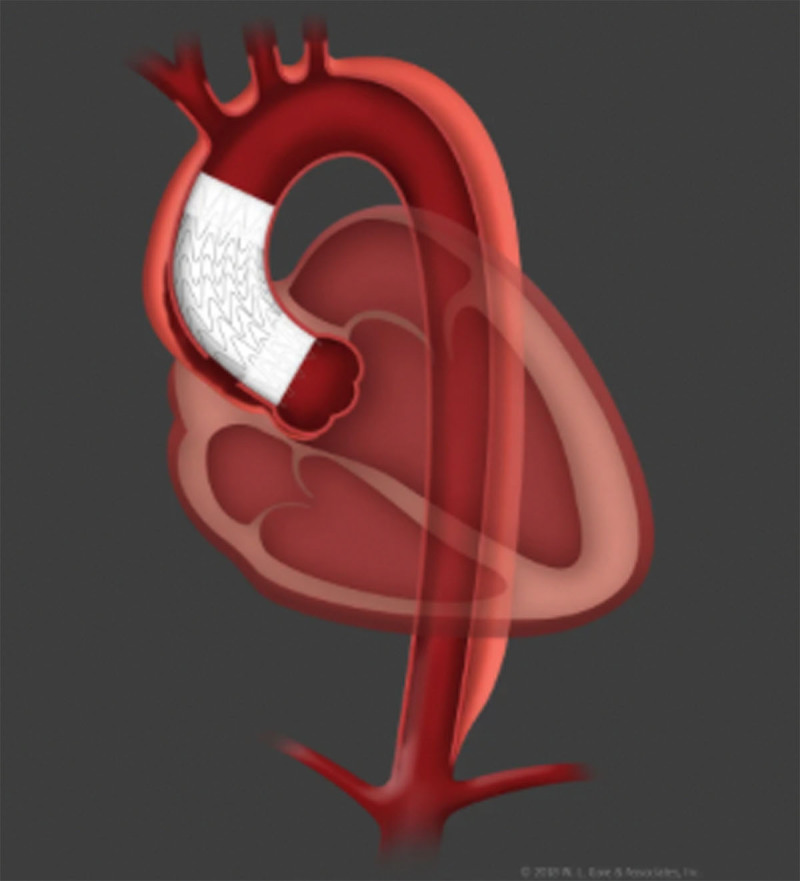
Gore Ascending Stent graft implanted for type A aortic dissection. Reprinted with permission. GORE^®^ ASCENDING STENT GRAFT © 2023. See Instructions for Use for complete device information, including approved indications and safety information.

The first thoracic stent graft designed specifically for the ascending aorta was recently reported in an early feasibility trial for the treatment of acute type A aortic dissection in patients considered at high risk for open surgery.^2^ This multicenter, prospective, nonrandomized, single-arm study enrolled 19 patients at seven of nine US sites. Nineteen patients were enrolled with a mean age of 75.7 years (range 47–91 years) and 11 (57.9%) were female. Ten (52.6%) had DeBakey type I disease, and the rest had type II. Sixteen (84.2%) of the patients were acute. Patients were treated with safe access—with 7/19 (36.8%) percutaneous, 10/19 (52.6%) transfemoral, and 2/19 (10.5%) iliac conduit)—and delivery/deployment was completed in all cases. The median procedure time was 154 minutes (range 52–392 min) and median contrast used was 111 mL (range 75–200 mL). Major adverse cardiovascular events at 30 days occurred in five patients, including mortality in 3/19 (15.8%), disabling stroke in 1/19 (5.3%), and myocardial infarction in 1/19 (5.3%). These results compare favorably to those seen in open surgical repair for type A aortic dissection and in a group of patients who were deemed high risk for open repair. A phase 2 study, expanded to additional sites, is planned for 2023. The Gore ascending stent graft device delivery system has the ability to first deploy to 50% and then articulate and angulate the device along the inner curvature, and it can then be deployed and angulated further, if necessary, prior to complete detachment (Figure 5). The device also has been used with the Gore single-branch thoracic endoprosthesis stent to extend the distal seal zone into the Innominate artery.

#### Endovascular Bentall

**Figure 6:**
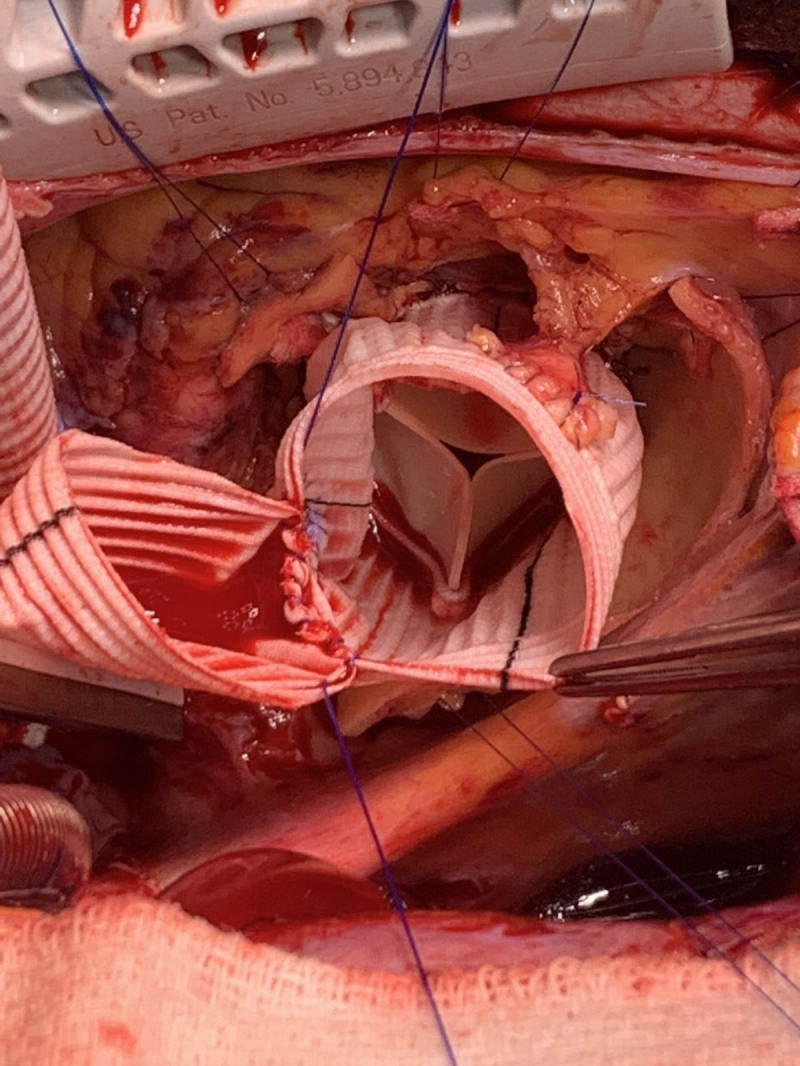
The Bentall procedure replaces aortic root and ascending aorta with reimplantation of the right and left coronary arteries as buttons.

One of the typical limitations to endovascular ascending aortic repair is the short proximal seal zone in the ascending aorta. The exact length needed for the seal remains to be determined but may be as short as 1 cm. In the setting of an ascending aortic dissection, the entry tear and dissection frequently extends into the aortic root, which would preclude a proximal seal zone. Current open surgical management in those deemed candidates would include a Bentall procedure (Figure 6), which involves replacing the aortic root and ascending aorta with a Dacron graft and reimplanting the right and left coronary arteries onto the graft as buttons. The concept of an endo-Bentall procedure has been discussed since it was first studied by Rylski et al. in 2014.^17^ The authors of this study postulated a transapically implanted endovascular device with a connected TAVR valve, which was attached to a proximally uncovered stent graft in the aortic root and a covered portion beginning at the sinotubular junction. This concept allowed for aortic valve stabilization and free flow into the coronary arteries but did not address any dilatation within the aortic root.

In 2020, Gaia et al. reported the first in human implantation of a custom endovascular Bentall device.^18^ The patient was a 64-year-old woman who had a previously placed bioprosthetic aortic valve for severe aortic stenosis and was subsequently lost to follow-up. She developed a pseudoaneurysm at the aortotomy site and had developed intermittent bleeding from a skin lesion in the suprasternal notch over the ensuing 2 years, but she had not undergone medical evaluation. Computed tomography angiography revealed a porcelain aorta and a pseudoaneurysm that had fistulized to the skin at the suprasternal notch. The patient had evidence of bioprosthetic structural valve degeneration with recurrent aortic stenosis, but she was not considered a candidate for redo open surgery. A custom-made device was designed, including a balloon expandable TAVR valve connected to a self-expanding aortic stent graft. The device was brought through the LV apex after a limited thoracotomy. The stent graft had two internal/external branches for the coronary arteries, which were then bridged with covered stents into the right and left main coronary ostia. The implantation of the covered stent grafts into the coronary ostia was performed with extracorporeal membrane oxygenation support. The patient was subsequently discharged to home 7 days post operatively, and follow-up CT revealed exclusion of the pseudoaneurysm; she was alive 9 months following the procedure without complication.

## Conclusion

The endovascular aortic revolution will continue to expand into the ascending aorta and aortic root in the foreseeable future. However, there remains much work to be done, both from a biomedical engineering device development standpoint and from a clinical trials perspective. Devices designed specifically for the ascending aorta are desperately needed for those patients not considered candidates for open surgical repair. Placing a non-expansile thoracic stent graft within the ascending aorta mitigates the anatomic Winkessel effect and places additional strain on the LV as well as the downstream aorta. The long-term effects of this are unknown regarding adverse LV remodeling or aneurysmal degeneration of the remaining segments of the aorta. The development of an endovascular Bentall solution to pathology involving the aortic root adds further complexity to an already difficult space. Given the paradigm shift prompted by endovascular stent grafts in the descending thoracic aorta, it would be logical to assume that further advancements in technology will allow endovascular stent grafting of the ascending aorta and root to become commonplace in the future, even for lower-risk patients.

## Key Points

- Endovascular management of pathologies of the ascending aorta is currently in its infancy but is desperately needed for patients not fit for open surgical repair.
- Devices designed specifically for use in the ascending aorta are currently in clinical trials or are in development.
- The first early feasibility clinical trial of an ascending specific device for use in the management of acute type A aortic dissection in high risk patients shows promising results in a highly select group of patients.
